# Smartphone-based thermography to determine shunt patency in patients with hydrocephalus

**DOI:** 10.1007/s13760-023-02338-3

**Published:** 2023-08-10

**Authors:** B. S. Harhangi, I. Voigt, N. Damee, P. S. Gadjradj

**Affiliations:** 1 Department of Neurosurgery, Erasmus MC, Dr. Molenwaterplein 40, 3000 CA Rotterdam, The Netherlands; 2 Department of Neurosurgery, Park MC, Hoofdweg 90, 3067 GH Rotterdam, The Netherlands; 3 Department of Pain Medicine, Erasmus MC, Dr. Molewaterplein 40, 3000 CA Rotterdam, The Netherlands

**Keywords:** Shunt, Hydrocephalus, Thermography smartphone

## Abstract

**Background:**

When shunt dysfunction is suspected, radiation exposure due to X-rays or a CT-scan is inevitable. Less-invasive and more reliable methods are warranted. In this study, we aimed to assess the usability of smartphone-based thermography to detect shunt patency in patients with hydrocephalus.

**Methods:**

This prospective observational pilot study evaluated the use of smartphone-based video thermography to detect flow of cerebrospinal fluid in the shunt of 51 patients from the Department of Neurosurgery at a tertiary referral institute. Patients with a shunt for hydrocephalus without the suspect of dysfunction were included in the study from December 2021 to May 2022.

**Results:**

We included 51 patients with a mean age of 53.3 years. Of these patients 14 were male (27.5%) and 37 were female (72.5%). The most frequent cause of the hydrocephalus was the normal pressure hydrocephalus followed by the congenital hydrocephalus. Most patients (96%) had a ventriculoperitoneal shunt, whereas two had a ventriculo-atrial shunt. In total, 43 patient (84%) had a shunt on the right side and 8 patient (16%) had the shunt located on the left side. In 45 patients (88.2%), we observed a clear flow of cerebrospinal fluid in the cooled shunt trajectory.

**Conclusions:**

The findings of this study indicate that in patients with a shunt to treat hydrocephalus, the smartphone-based video thermography may be a safe and simple alternative to show shunt patency without the exposure to radiation.

## Introduction

Hydrocephalus is a disorder caused by an abnormal buildup of cerebrospinal fluid (CSF) in the ventricles of the brain. Ventriculoperitoneal shunts (VP-shunt) are the most common treatment option for hydrocephalus. The shunt is used to circumnavigate an obstruction or to drain excess CSF. Usually, the shunt consists of three parts: the proximal catheter, a one-way valve, and a distal catheter [1]. Shunt complications, however, are a common problem in these patients; they can be a result of a dysfunctional shunt, mechanical failure, or infection [7]. Options to detect shunt dysfunction include an X-ray of the shunt system and a cranial Computer Tomography-scan (CT-scan). An important disadvantage, apart from the costs, is the exposure to radiation; especially in children and fertile women [3]. Furthermore, CT-scans without findings for valve dysfunction, including normal or small size of the ventricles, could also be false-negative. An alternative for the CT-scan may be the magnetic resonance imaging, but this is more time-consuming and costly, and may also not help solve this diagnostic problem.

Another alternative for detecting shunt dysfunction is shunt tapping which is an invasive procedure associated with an increased risk of infections and the possibility of damaging the shunt. Since these methods are associated with their own drawbacks and the cumulative exposure to radiation in fertile women and children, there is a clinical need for a simple, noninvasive adjunct with minimal to no radiation exposure to detect shunt dysfunction. In the past, the ability to assess shunt flow patency with video thermography was investigated [4–6]. It has been shown that the FLIR (forward looking infrared) One camera is a reproducible method to assess the pattern of skin temperatures in healthy individuals [2]. Hence, the presented method using a smartphone with a thermal imaging camera may be a simpler and faster tool to detect shunt flow without the exposure to radiation. Therefore, the objective of this study was to determine if FLIR One is able to detect flow of CSF in patients with no clinical signs of shunt dysfunction.

## Methods

From December 2021 to May 2022, consecutive patients with a shunt from the outpatient clinic at the Department of Neurosurgery at our institute were included in the study. We used the second-generation FLIR One video camera, an I-phone 6 SE with IOS 14, and a cold-pack to detect shunt flow.

Inclusion criteria were patients with a shunt implanted to treat hydrocephalus, visiting the outpatient clinic for a regular checkup without suspicion of shunt dysfunction. Exclusion criteria included pregnancy, tumors, lack of cooperation of the patient, and patients who were not mentally able to complete the questionnaires, according to the referring doctor and patients under the age of 18.

**Fig. 1:**
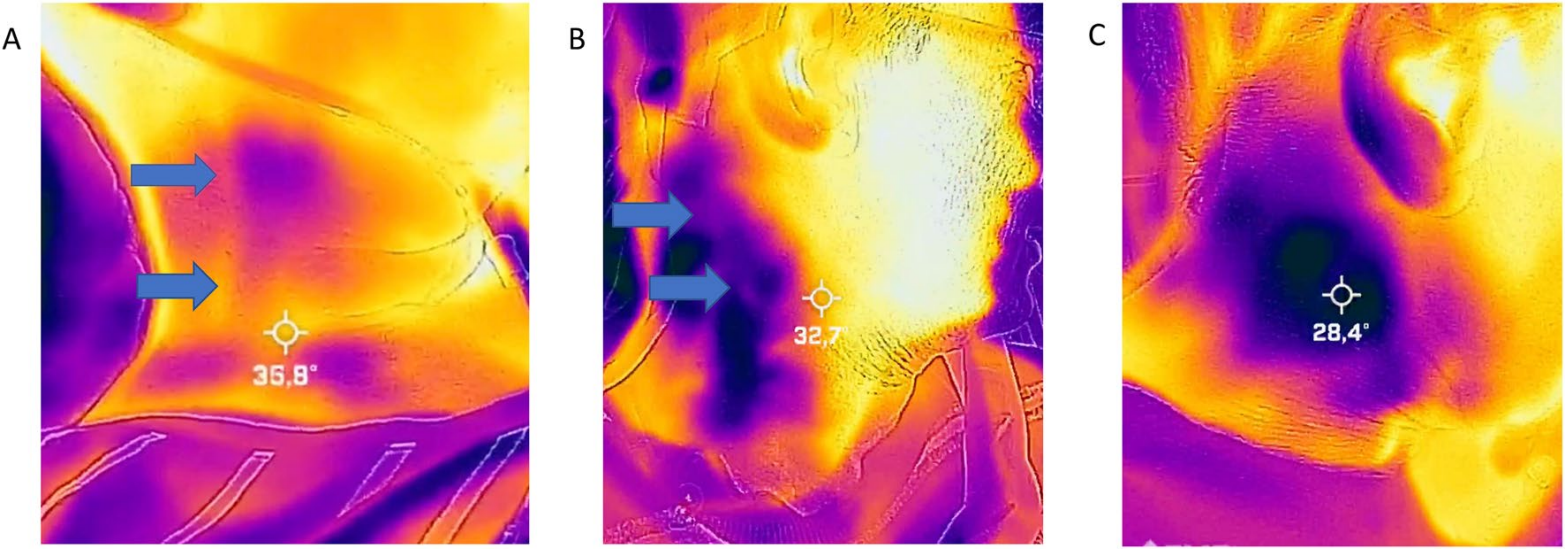
**A** Image of the smartphone-based video thermography in a patient with hydrocephalus without signs of shunt dysfunction; **B** Image in which the flow was doubtful and **C** No signs of flow. Flow in the shunt, if any, is marked by the blue arrows (Color figure online)

To make the CSF flow visible on the FLIR One camera, a cold-pack was placed for a minute on a location below the valve where we could feel the shunt manually. After applying the cold-pack for 1 min, the cooled location, and the location underneath (distal), the cooled location was recorded for 10 min with the FLIR One video camera. The acquired video images and results gathered from the cooled location and the location distal to the cooled location were used to observe the temperature-related position of the shunt and the 10 min time-related flow test. All videos were visually examined by two reviewers simultaneously to reach consensus. After removing the coldpacks, the cooled area turned into blue color indicating low temperatures, and the surrounding area was yellow indicating normal skin temperature. Shunt flow was defined as patent if a clear yellow line was seen in the blue colored cooled area, indicating higher temperature due to traversing warm CSF in the shunt (Fig. 1A). If the line was not clearly visible than it was scored as doubtful (Fig. 1B), and if no color differences were observed around the shunt trajectory, it was scored as not visible (Fig. 1C). The study was approved by our Medical Ethical Committee and all patients have given written informed consent for the study.

### Data analysis

All images were analyzed using FLIR One tools and software. This is software which is enclosed with the camera with the free available FLIR ONE software in the Apple app store. The temperature was measured using the FLIR One camera. Flow was visually judged by discoloration of the shunt trajectory color. Data were analyzed using SPSS (version 25.0).

## Results

In this prospective observational study, we included 51 patients with a mean age of 53.3 years. Of these patients 14 were male (27.5%) and 37 were female (72.5%). Table 1 summarizes the patients’ characteristics, shunt characteristics, and observed shunt flow. The most frequent cause of the hydrocephalus was the normal pressure hydrocephalus followed by the congenital hydrocephalus. Most patients (96%) had a ventriculoperitoneal shunt, whereas two had a ventriculo-atrial shunt (VAS). In total, 43 patients (84%) had a shunt on the right side and 8 patient (16%) had the shunt located on the left side. The Certas valve was most frequently observed valve (52.9%) followed by the Strata valve (19.6%). The mean revision rate in the past was 2.3. Ten patients (19.6%) had a recent revision, i.e., within the previous 7 days. Figure 1A shows the FLIR One image where the yellow line suggests flow in the shunt is shown by the blue arrow. In Fig. 1B, we see some color changes from blue to yellow but not as clear as in 1A. In Fig. 1C, no color differences were observed within the 10 min after cold-pack removal. In 45 patients (88.2%), we observed a clear yellow line in the cold blue area indicating flow in the cooled shunt trajectory, whereas in 5 patients (9.8%), we had some doubt about the flow, and in one patient (2%), there was no flow visible at all but without symptoms of shunt dysfunction.

**Table 1:** Patient and shunt characteristics

Patients’ characteristics (*n* = 51)	
Male	14 (27.5%)
Female	37 (72.5%)
Age (years)	53.3 (± 18.4)
Weight (kg)	76.2 (± 1.5)
Length (m)	1.69 (± 0.1)
Causes of hydrocephalus	
Normal pressure hydrocephalus	17 (33.3%)
Congenital	10 (19.6)
Subarachnoid hemorrhage	8 (15.7%)
Aqueduct stenosis	3 (5.9%)
Post-infection	1 (2.0%)
Other	12 (23.5%)
Shunt type	
Certas	27 (52.9%)
Strata	10 (19.6%)
Delta	6 (11.8%)
PS medical	5 (9.8%)
Mietke	1 (2.0%)
Ventriculo-atrial shunt	2 (4.0%)
Shunt flow	
Visible	45 (88.2%)
Doubtful	5 (9.8%)
Not visible	1 (2.0%)

## Discussion

In this study, we are the first to report on the use of the smartphone-based FLIR One thermal imaging camera to detect shunt flow in patients without a suspect of proximal shunt dysfunction. The observation of the temperature-related differences in the cooled area were clear to show flow over the shunt. In 88% of the patients with no suspect of shunt dysfunction, we could observe flow in the cooled trajectory. These results may be useful in decision-making and could be a widely available, screening tool for the general practitioner or lower middle-income countries. In the study of Goetz et al. [4], they used the thermography to detect cold CSF in the shunt from the proximal cooled area, whereas we tested for warm CSF in the cooled area. In 85% of the patients, Goetz et al. could observe CSF flow in the shunt. In this study, we observed comparable CSF flow in the shunt in (88%). Different parameters, including type of hydrocephalus, gender, hemoglobin level, electrolyte values, and differences between patients’ body mass index, are factors that may affect the results and explain why we did not see flow in all patients. To the best of our knowledge, FLIR One’s reliability in assessing shunt dysfunction has not been evaluated yet. Infrared thermography has a high sensitivity and specificity when it comes to detecting brain blood flow changes [9]. It has also been shown that real-time evaluation of cerebral blood flow in arteriovenous malformations or patency of bypass is possible with infrared thermography [8, 9]. In patients with a hydrocephalus, it may be a potentially simple, safe, and cost-effective mobile adjunct to X-rays and CT-scans.

### Strengths and limitations

One of the strengths is that the smartphone-based FLIR One camera is a relatively simple tool to use and inexpensive resources are needed to apply it. Another strength is that we observed a specificity of 88%, suggesting that this test may be useful in diagnosing shunt patency.

Limitations include the lack of reproducibility. No validated techniques are available yet, and hence, a certainly subjective visual observational scoring was used to assess shunt flow. Another limitation is that no data on reliability and sensitivity are available yet. For example, we do not know what the flow is like in partial shunt dysfunction. As with any new technique, it should be compared to the already established methods. Other limitation ar that this data set does not cover pediatric patients as well as the fact that BMI in this study is rather low with an average weight of 76.2 kg (CI ± 1.5). Finally, data on flow velocity and clinical shunt patency were not investigated but very important to understand the relation between shunt dysfunction and shunt flow. Future studies must focus on the clinical reliability of smartphone-based thermography in comparison to the existing diagnostic tools, including X-rays, CT-scans, and MRI scans in patients with patent and non-patent shunt function.

## Conclusion

In patients with a CSF shunt, a smartphone-based thermography camera may be a safe and simple adjunct to show shunt patency. Future studies should focus on sensitivity and reliability of this method in patients with patent and non-patent shunt function.

## Data Availability

Upon request data is available.

## Abbreviations

CSF: Cerebrospinal fluid
CT: Computer tomography
FLIR: Forward looking infrared
VP: Ventriculoperitoneal
VAS: Ventriculo-atrial shunt
MEC: Medical ethical committee
IT: Infrared thermography
